# Supplementary material for: #LetsUnlitterUK: A demonstration and evaluation of the Behavior Change Wheel methodology
Source: PLoS One. 2021 Nov 16;16(11):e0259747. doi: 10.1371/journal.pone.0259747 (PMC8594830; doi:10.1371/journal.pone.0259747)
Supplement: S1 Appendix — (DOCX) [file pone.0259747.s001.docx]

**Appendix A**

Each domain (and its definition; Cane et al. 2012), and its associated survey items.

| Theoretical domain  (definition) | Diagnostic Survey 1 | Diagnostic Survey 2 |
| --- | --- | --- |
| Knowledge (An awareness of the existence of something) | - I know how to write a post on Twitter. | - I know how to write an anti-littering post on Twitter. |
|  | - I know how to post a picture on Twitter. - I know what is the objective of posting anti-littering messages on Twitter.* | - I know how to post an anti-littering picture on Twitter. |
| Skills (An ability or proficiency acquired through training and/or practice) | - I have experience posting on Twitter. | - I have experience posting on Twitter. |
|  | - I have the skills necessary to post anti-littering messages on Twitter. | - I have the skills and ability necessary to post anti-littering messages on Twitter. |
| Memory, attention, and decision processes (The ability to retain information, focus selectively on aspects of the environment and choose between two or more alternatives) | - I often intend to post an anti-littering message on Twitter and then forget to do it. |  |
|  | - There often are distractions online, or around me, which prevent me from posting anti-littering messages on Twitter. ^(RS)^ | - I usually have no distractions to prevent me from posting anti-littering messages on Twitter. |
|  | - It is easy for me to select an anti-littering topic to Tweet about.* | - It is easy to remember to post anti-littering messages on Twitter. |
| Behavioral regulation (Anything aimed at managing or changing objectively observed or measured actions) | - Posting anti-littering messages on Twitter is something I would automatically, without thinking. | - I would post anti-littering tweets automatically, without even thinking about it. |
|  | - I can keep track of my overall progress in posting anti-littering messages on Twitter, e.g. by keeping track of the number of posts I tweet. | - I could keep track of how much I use Twitter to post anti-littering messages. |
| Social influences (Those interpersonal processes that can cause individuals to change their thoughts, feelings, or behaviors) | - Most people who are important to me would think it is a good idea for me to post anti-littering messages on Twitter. | - Most people would think it is a good idea for me to post anti-littering messages on Twitter. |
|  | - I know other people who post anti-littering messages on Twitter. | - I know other people who post anti-littering messages on Twitter. |
| Environmental context and resources (Any circumstance of a person’s situation or environment that discourages or encourages the development of skills and abilities, independence, social competence, and adaptive behavior) | - I regularly use a smart phone. | - I regularly use a smart phone. |
|  | - Often there are times, for example at work or in school, when I have no access to Twitter.^(RS)^ | - Most of the time I have access to Twitter. |
| Social/professional role and identity  (A coherent set of behaviors and displayed personal qualities of an individual in a social or work setting) | - As an influential person, it is my job to post anti-littering messages on Twitter. | - As an influential person, it is my job to post anti-littering messages on Twitter. |
|  | - Posting anti-littering messages on Twitter is consistent with who I am. | - Posting anti-littering messages on Twitter is consistent with who I am. |
| Beliefs about capabilities (Acceptance of the truth, reality or validity about an ability, talent, or facility that a person can put to constructive use) | - I can post anti-littering messages on Twitter even if other people are not motivated to do so. | - I can post anti-littering messages on Twitter even if other people are not motivated to do so. |
|  | - I can post anti-littering messages on Twitter even if I have little time. | - I can post anti-littering messages on Twitter even if I have little time. |
| Optimism (The confidence that things will happen for the best or that desired goals will be attained) | - I feel optimistic about the impact that posting anti-littering messages on Twitter can have. | - I feel optimistic about the impact that posting anti-littering messages on Twitter can have. |
|  |  | - I expect posting an anti-littering message to have a positive impact. |
| Intentions (A conscious decision to perform a behavior or a resolve to act in a certain way) | - I will definitely post an anti-littering message on Twitter in the next seven days. | - Within the next week I intend to post an anti-littering message on Twitter. - I will definitely post an anti-littering message on Twitter in the next seven days. |
| Goals (Mental representations of outcomes or end states that an individual wants to achieve) | - I have an idea of what anti-littering messages I will post on Twitter. | - I know the type of anti-littering messages I will post on Twitter. |
|  | - I know under what circumstances I will post anti-littering messages on Twitter. | - I know under what circumstances I will post anti-littering messages on Twitter. |
|  | - Posting anti-littering messages on Twitter is often less urgent than doing other things.* ^(RS)^ |  |
| Beliefs about consequences (Acceptance of truth, reality, or validity about outcomes of a behavior in a given situation ) | - Posting anti-littering messages on Twitter can make a change. | - Posting anti-littering messages on Twitter can make a change. |
|  | - Posting anti-littering messages on Twitter will have a positive effect on people in my social network. | - Posting anti-littering messages on Twitter will have a positive effect on people in my social network. |
| Reinforcement (Increasing the probability of a response by arranging a dependent relationship, or contingency, between the response and a given stimulus) | - Having my anti-littering Tweets retweeted, or liked, by other people would motivate me to post even more such messages. | - I am motivated to post anti-littering messages by the recognition I would get from people who are important to me. |
|  | - I would get recognition from people who are important to me if I posted anti-littering messages on Twitter. | - I am motivated to post anti-littering messages on Twitter by the retweets I might earn. |
|  | - Getting monetary rewards, or other incentives, for posting anti-littering messages on Twitter would motivate me to do it more often.* |  |
| Emotions (A complex reaction pattern, involving experiential, behavioral and physiological elements by which the individual attempts to deal with a personally significant matter or event) | - Posting anti-littering messages on Twitter would make me feel good. | - Posting anti-littering messages on Twitter would make me feel positive. |
|  | - Posting anti-littering messages on Twitter is boring or annoying.^(RS)^ | - Posting anti-littering messages on Twitter would make me feel good. |

* Indicates the statement removed to increase the reliability of Diagnostic Survey 1’s Knowledge, Memory attention, and decision processes, Goal and Reinforcement scales

^(RS)^ indicates a reverse scored item
